# Associations between Psychosocial Working Conditions and Quality of Care (i.e., Slips and Lapses, and Perceived Social Interactions with Patients)—A Cross-Sectional Study among Medical Assistants

**DOI:** 10.3390/ijerph18189693

**Published:** 2021-09-15

**Authors:** Viola Mambrey, Patricia Vu-Eickmann, Peter Angerer, Adrian Loerbroks

**Affiliations:** Centre for Health and Society, Faculty of Medicine, Institute of Occupational, Social and Environmental Medicine, University of Düsseldorf, Moorenstr. 5, 40225 Düsseldorf, Germany; viola.mambrey@uni-duesseldorf.de (V.M.); Patricia.Vu-Eickmann@uni-duesseldorf.de (P.V.-E.); peter.angerer@uni-duesseldorf.de (P.A.)

**Keywords:** errors, health care staff, medical assistants, psychosocial working conditions, quality of care, slips and lapses, social interaction, work stress

## Abstract

Adverse psychosocial working conditions in the health care sector are widespread and have been associated with a reduced quality of patient care. Medical assistants (MA) assume that their unfavorable working conditions predominantly lead to a poorer quality of care in terms of slips and lapses, and poorer social interactions with patients. We examined those associations for the first time among MAs. A total of 944 MAs in Germany participated in a survey (September 2016–April 2017). Psychosocial working conditions were measured by the effort-reward imbalance (ERI) questionnaire and a questionnaire specifically designed for MAs. Slips and lapses (3 items, e.g., measurement or documentation errors) and the quality of interactions (3 items) with patients were measured by a questionnaire developed by the study team based on prior qualitative research. We ran Poisson regression to estimate multivariable prevalence ratios (PRs). The ERI ratio and MA-specific working conditions were significantly associated with frequent self-reported slips and lapses (PR = 2.53 and PR ≥ 1.22, respectively) or poor interactions with patients (PR = 3.62 and PR ≥ 1.38, respectively) due to work stress. Our study suggests that various types of adverse psychosocial working conditions are associated with perceptions of slips and lapses or poorer interaction with patients due to work stress among MAs.

## 1. Introduction

A high workload [1,2,3], low job control [1,2,3], poor organization of work processes [2,3] as well as a lack of social support [3,4] and resources [3] are amongst the adverse psychosocial working conditions that healthcare workers face in their everyday work. Such unfavorable psychosocial working conditions are associated with stress [5,6,7]. Work stress in turn is associated with poor mental and physical health [8,9]. Moreover, numerous studies suggest that adverse psychosocial working conditions [10,11,12], the perception of work stress [11,12,13], and mental health (e.g., burnout) [14] in health care staff are associated with poorer patient care as indicated, for instance, by increased rates of medical errors or lower ratings of the quality of care as provided by patients or the health care workers themselves.

In Germany, outpatient care is mainly provided by general physicians (GPs) or specialists and their medical assistants (MAs). MAs represent the largest occupational group in outpatient care in Germany [15]. They perform a wide range of tasks in the practice, including administration, management, documentation, and to some extent medical procedures (e.g., blood collection, injections, wound care, X-rays, and laboratory diagnostic). In addition, MAs are the first point of contact for patients and are therefore responsible for assessing the urgency of patients’ medical concerns, also known as triage, and need to schedule appointments accordingly [3,16]. Further, MAs often establish and maintain long-term and close social relationships with patients and thereby ensure patients’ retention to the practice, which represents an activity that differs from the set of tasks of health care professionals in hospital settings [17,18]. All those tasks are closely related to the quality of patient care, which MAs perform often as delegated by their supervising physician. Moreover, outpatient care is usually characterized by working in small teams and the fact that the physician often fulfills the position of both the employer and supervisor [1]. These differences in tasks and settings imply multiple profession-specific stressors among MAs (e.g., practice organization, social stressors, or task variation) [3] compared to other health professions, such as nurses in hospitals or physicians in outpatient care. Psychosocial working conditions of MAs have repeatedly been found to be poor and their chronic stress level to be particularly high [5,11].

In prior work among MAs, we observed positive associations of various types of adverse psychosocial working conditions with MAs’ concerns to have made important errors and the perceived interference of work stress with patient care [11]. However, in a previous qualitative study, we also found that MAs seem to perceive the greatest impact of their working conditions on slips and lapses, i.e., minor errors related to inattentiveness (e.g., documentation errors or measurement errors) and poor social interactions with patients rather than on important or major errors [19]. This is in line with a study by Gehring and Schwappach [16] in which practice staff reported that the most frequent type of error was documentation errors. Notably though, such allegedly minor errors can have major implications for patients’ health and safety. Slips and lapses related to documentation or measurements on the patient could lead to misdiagnoses and incorrect prescriptions, which are frequent in primary care [20]. Furthermore, with regard to triage, for instance, it has been shown that difficulty related to the communication with patients can lead to misinterpretations of the health status and possibly result in major adverse health outcomes among patients, such as hospitalization or death [16,21]. In addition, it has been suggested that for patients, a successful helpful depression case management depends on a good social interaction in terms of trust with MAs [18]. These examples support the notion that the prevention of slips and lapses as well as appropriate social interaction are crucial for the delivery of optimal patient care.

Studies among healthcare professions have investigated the association between psychosocial working conditions and quality of care indicators, focusing mainly on physicians in the outpatient sector or on staff in hospital-based settings [4,10,12,22]. Nevertheless, it seems plausible that associations of psychosocial work conditions with the quality of care vary among different types of health care professions (e.g., physicians, nurses, MAs) due to differing stressors and tasks (see above). This potentially limited generalizability of findings across professions warrants empirical studies for different types of health care professions. So far, among MAs, there is no scientific evidence related to the potential links between psychosocial working conditions and the quality of care in terms of slips and lapses as well as perceived social interactions with patients. We aimed to address this gap by quantifying those associations for the first time to inform the development of preventive measures to increase the quality of patient care provided by MAs.

## 2. Materials and Methods

### 2.1. The Study Sample

Data of 944 MAs were collected either by an online survey or postal survey in line with respondents’ preferences (September 2016–April 2017). There were no specific inclusion or exclusion criteria; thus, all MAs were eligible. Recruitment of participants was carried out nationwide in Germany and with the support of various associations and organizations and multiple communication channels. Amongst others, we included flyers in the members magazine of the Association of Medical Professions (VMF e.V.), which represents MAs. We further advertised our study at various regional Associations of Statutory Health Insurance Physicians via internal distribution, home pages, or direct forwarding to medical practices or professional MA schools. Recruitment of medical assistants was also carried out by presenting our study at relevant conferences and training events (see acknowledgements for details). Due to the widespread recruitment, the response rate cannot be estimated (i.e., unknown denominator). While the full scope of the sample’s representativeness remains uncertain, it deserves mentioning that our study population matches the overall MA population in Germany well in terms of age, gender, and employment status, as estimated by the Federal Statistical Office [15]. In addition, in another study among GPs and MAs, including a representative sample of GP practices in Germany, MAs’ characteristics were comparable to those of our study population [5]. These included age, number of persons in household, marital status, years of work experience, and employment status. Our study was approved by the Ethics Committee of the Medical Faculty of the Heinrich–Heine University of Düsseldorf (ethic registration number: 4778). When we wrote this report, we drew on the STROBE (STrengthening the Reporting of Observational studies in Epidemiology, see: https://www.strobe-statement.org/, accessed on 13 July 2021) guidelines whenever feasible.

### 2.2. Survey Instruments

#### 2.2.1. Psychosocial Working Conditions

Psychosocial working conditions were measured by two instruments, i.e., the theory-based effort-reward imbalance (ERI) questionnaire [7] and a questionnaire capturing working conditions specifically as experienced by MAs [11]. The ERI model postulates that a state of emotional distress is elicited by a combination of high efforts spent (i.e., high workload, time pressure, or responsibility) and low reward received (i.e., low salary, low esteem, poor career prospects). The ratio between effort and reward is used to capture the imbalance of the two components at the individual level [7]. In this study, we applied the validated ERI questionnaire that measures psychosocial working conditions by 17 items [7]. The items are presented as statements and are answered on a 4-point Likert scale with the level of agreement ranging from “I strongly disagree” (1) to “I strongly agree” (4) and can be combined into the subscale effort (6 items, potential score range = 6 to 24; Cronbach’s alpha (CA) in this study = 0.60) and the subscale reward (11 items, potential score range = 11 to 44; CA in this study = 0.82). Increasing scores indicate a higher level of agreement to the respective subdimension. To derive the ERI ratio, the sum-scores of the subdimensions effort and reward are calculated and weighted by the number of items of the opposing subdimension. As recommended [7], the scoring of some items was reversed when the reward sum score was calculated. The ERI ratio reflects the imbalance between effort and reward, with an ERI value >1.0 indicating work stress. A high ERI ratio is associated with adverse physical and mental health outcomes [8,23].

The MA-specific questionnaire [11] aimed to capture relevant day-to-day working experiences of MAs [3] and was developed based on a multi-stage process, i.e., a qualitative study, cognitive interviews with Mas, and psychometric analyses [3,11]. The MA-specific questionnaire measures seven types of psychosocial working conditions, which are captured by 3–6 items each (29 items in total). Items are presented as statements. Responses are provided on a 4-point Likert scale (varying from “I fully agree” to “I fully disagree”) and are added across the respective factors to drive factor-specific sum scores. The factors are as follows: (1) workload (e.g., overtime, time pressure, number of patients, and staff shortage; 6 items; CA = 0.85, potential score range = 6–24); (2) job control (e.g., documentation effort and unforeseen events, interruptions, and multitasking; 6 items; CA = 0.77, potential score range = 6–24); (3) collaboration with supervisor/colleagues (e.g., working climate and treatment by others; 4 items; CA = 0.74, potential score range = 4–16); (4) gratification (e.g., career prospects and recognition; 4 items; CA = 0.71, potential score range = 4–16); (5) practice organization (e.g., work structure and responsibilities; 3 items; CA = 0.78, potential score range = 3–12); (6) resources (e.g., interaction with patients and variety of work tasks; 3 items; CA = 0.72, potential score range = 3–12); and (7) leadership behavior (e.g., recognition and work organization; 3 items; CA = 0.74, potential score range = 3–12). The CA for the entire 29-item questionnaire was 0.89. To harmonize the interpretation of the answers, three items of factor 4 and all items of factors 5–7 were reversed when sum scores were calculated. A higher score consistently indicates a higher stressor exposure.

Our approach to combine the ERI questionnaire and our MA-specific questionnaire into a single study draws on the advantages of each type of instrument while minimizing the respective weaknesses: the ERI questionnaire is well-established and widely used in a standardized format. As such, the ERI allows for the comparison of findings across different studies and populations. However, as shown above, the CA of the effort scale was rather low in our study population (0.60). This relatively low CA suggests that MAs do not agree to an equal extent for each item of the effort scale. This may imply that the effort scale captures efforts whose relevance varies for MAs. Thus, at the cost of its standardization and its applicability across all occupational subpopulations, the ERI may fail to capture the full range of important adverse psychosocial working conditions as experienced by MAs (i.e., also measuring less relevant conditions). For instance, with regard to MAs, we found various additional stressors (e.g., unforeseeable events, multitasking, and poor collaboration with colleagues and supervisor) and resources (e.g., good practice organization, task variation, and the social aspects of the work) [3]. All these factors are covered by the MA-specific questionnaire, which thus has the advantage of high content validity but at the cost of limited applicability to other professional populations.

#### 2.2.2. Quality of Care

Quality of care was measured by a questionnaire, which was newly developed by the study team, as a suitable questionnaire was unavailable. We drew on insights into the influence of work stress on the quality of care, as perceived by MAs, from our prior qualitative work [19] to develop this questionnaire. In brief, qualitative interviews with MAs revealed that MAs perceived that work stress affects the quality of care they provide primarily in terms of poorer social interactions with patients and an increase of slips and lapses, i.e., minor errors due to carelessness (e.g., documentation errors, measurement errors) [19]. Based on the qualitative interviews, items were developed by the study team to capture relevant facets of poor interactions and slips and lapses. The questionnaire was refined by means of cognitive interviews, which explored the overall impression, comprehensibility, completeness, and suggestions for revision. The final instrument comprises six items that are presented as statements. Three items capture the perceived frequency of slips and lapses due to work stress while working with the patient, with patient records or on documentation. The three remaining items reflect the frequency of difficulties in social interactions with patients due to stress in terms of unfriendliness, impatience, and perceived time pressure. Each item is scored using a 5-point Likert scale (never = 1; rarely = 2; occasionally = 3; most of the time = 4; always = 5), with higher scores reflecting participants’ agreement that work stress affects the respective aspect of care. Exploratory factor analyses (VARIMAX rotation) suggested separation of the items into two factors consisting of three items each (see Table 1). The factors were named “slips and lapses” and “poor interaction with patients,” respectively, and each had a possible range of values from 3 to 15 points. CA was 0.81 for the subscale “slips and lapses” and 0.69 for “poor interaction with patients”.

### 2.3. Statistical Analysis

Exposures were operationalized in terms of both dichotomized and continuous variables (z-scores). The ERI ratio was dichotomized according to its theory-based cut-off (>1.0 vs. ≤1.0). In keeping with previous research [11,13,25], we decided to apply distribution-based cut-offs to all other exposure variables (i.e., effort, reward, and the seven subdimensions of the MA-specific instrument), which were dichotomized based on the respective highest tertile (vs. the remaining tertiles). With regard to the outcome variables, however, the distribution of the variable “slips and lapses” did not allow splitting the sample into tertiles. In order to apply a cut-off that is still meaningful and statistically efficient, we decided to employ a distribution-based cut-off capturing the top 20% (e.g., a score of ≥7). The alternatives were a cut-off at ≥6, which represents roughly the median and has limited meaningfulness. A cut-off at ≥8, by contrast, categorized 88.3% of the sample in the reference group, which may lead to analytical difficulties (i.e., limited statistical power). The outcome variable “poor interaction with patients” was categorized using the same distribution-based cut-off (i.e., top 22.7%, i.e., a score of ≥10) to harmonize distribution-based cut-offs across outcomes.

Statistical analyses were performed using IBM SPSS 25.0. Missing values were not imputed. The percentage of missing values ranged from 0.45% (i.e., slips and lapses) to 5.86% (i.e., ERI-ratio). The primary statistical analysis was based on dichotomized exposures (e.g., ERI variables, MA-specific working conditions) and dichotomized outcomes (quality of care indicators). Associations were quantified by prevalence ratios (PRs) and corresponding 95% confidence intervals (95% CIs), which were estimated based on Poisson regression with robust estimator [26]. Poisson models were computed separately for effort, reward, ERI, and each of the subscales of the MA-specific questionnaire with one exception: the subscale “resources” of the MA-specific questionnaire was removed from our analyses with the outcome “social interaction with patients”. That subscale comprised items that seemed to conceptually overlap with the outcome “social interaction with patients” (these items were: “I enjoy the interaction with patients” and “I enjoy the fact that my profession is a social activity”). Similarly, the outcome “social interaction with patients” comprised an item capturing “time pressure”. Time pressure is also partially measured by the subscale “workload” of the MA-specific questionnaire (i.e., two out of six items) as well as the subscale “effort” of the ERI questionnaire (one out of six items) and the corresponding ERI-ratio. Thus, again, there may be some thematic overlap between these exposure variables and the outcome. In additional analyses, we therefore re-created the exposure subscales (i.e., workload, effort and ERI-ratio) while excluding the ”time pressure” items and re-ran the Poisson regression and linear regression analyses with the outcome “social interaction with patients”. Those analyses yielded similar results, though, (data not shown) compared to analyses with the original exposure scales.

We first ran unadjusted models (Model I) and then made adjustments for age, sex, and leadership position (Model II). We also carried out various types of sensitivity analyses to explore the robustness of our findings in light of differing analytical approaches. First, we also estimated PRs by Poisson regression using continuous exposures (i.e., z-scores). Second, we used linear regression models to analyze continuous outcome variables (i.e., z-scores) in combination with either dichotomized or continuous exposures.

## 3. Results

### 3.1. Sample Characteristics and Descriptive Results

In total, 944 MAs participated, and we limited the sample to those 887 MAs who reported to be in current employment as a MA. The average age of participants was 39.3 years, and the age range was wide (standard deviation (SD) = 11.4, see Table 2). As much as 98.4% were female, and 48.0% of the participants reported to hold a leadership position. Work stress in terms of the ERI ratio had a prevalence of 73.8%. In view of the potential score ranges, the mean value for effort was high, and reward was at an intermediate level. Comparing the mean values in light of the score range of each MA-specific factor, unfavorable working conditions were particularly pronounced in terms of a high workload, low job control, and poor leadership behavior. All exposure variables correlated with the outcomes “slips and lapses”, and “patient interaction” (see Table S1).

### 3.2. Primary Statistical Analyses

In our primary statistical analyses, we observed significant positive associations of working conditions with work stress-related slips and lapses as well as poor interaction with patients (see Table 3). With regard to slips and lapses, those with a high ERI score had more than a doubled prevalence of reporting a frequent link of slips and lapses with works stress compared to those with low ERI (PR = 2.53, 95% CI = 1.63–3.92). In case effort was high, slips and lapses in association with work stress were reported about 1.5 times more often (PR = 1.45, 95% CI = 1.10–1.90). For reward, an expected inverse relationship was observed (PR = 0.53, 95% CI = 0.38–0.75). The associations between the MA-specific working conditions and frequent perceived links between work stress and slips and lapses were significant with all PRs ≥ 1.39 except for the association between poor leadership behavior and the outcome (PR = 1.22, 95% CI = 0.92–1.61). Poor practice organization showed a particular pronounced association with self-reported frequent slips and lapses due to work stress (PR = 2.24, 95% CI = 1.73–2.90).

Overall, all adverse psychosocial working conditions were significantly associated with work stress-associated poor interactions with patients (see Table 3). Briefly, participants with a high ERI were 3.6-fold more likely to report a frequent link between work stress and poor interaction with patients than those with a low ERI (95% CI = 2.25–5.81). Likewise, effort and reward were associated with the outcome (PR = 1.88, 95% CI = 1.47–2.40 and PR = 0.42, 95% CI = 0.30–0.59, respectively). For MA-specific working conditions, consistent and positive associations of meaningful magnitude were observed with self-reported poor interactions with patients due to work stress (PRs ≥ 1.38). Particularly pronounced associations were found for a high workload, low job control, as well as poor collaboration (PRs = 2.0–2.3).

### 3.3. Sensitivity Analyses

Sensitivity analyses of continuous exposures (z-scores) showed similar patterns of associations (see Table 4). The linear regression models confirmed the findings from the primary analysis (see Tables S2 and S3).

## 4. Discussion

We found that a large set of psychosocial working conditions was associated with a perceived frequent impact of work stress on slips and lapses or poor social interaction with patients among MAs. Overall, associations were more pronounced with self-reported poor social interactions with patients than with slips and lapses. In terms of MA-specific work characteristics, particularly poor practice organization was found to relate to self-reported slips and lapses due to work stress. High workload, low job control, and poor collaboration showed fairly pronounced associations with both quality of care outcomes.

### 4.1. Comparison to Prior Research

In general, our results are consistent with the current evidence base; however, no study so far has focused on slips and lapses as well as poor social interactions with patients due to work stress and on the occupational group of MAs. Due to the different range and nature of stressors (see Section 1 (Introduction)), comparability of our findings with those from other health professions is limited.

High work stress according to the ERI model, reflecting an imbalance between the effort spent and the reward received (e.g., salary, recognition career prospects), has been associated with poor quality of care in terms of various different indicators (e.g., hospital infections, diabetes control, error frequency, self-assessed performance) [13,25,27,28]. It has been speculated, for instance, that a high ERI results in a lowered work motivation and a corresponding poorer commitment to patient safety protocols [27]. Studies relying on self-reported data among physicians found associations between a high ERI and increased errors as well as poorer patient care [13,25]. The afore-mentioned studies focused on rather severe errors, though, whereas our results expand the existing knowledge related to the ERI model to minor errors (i.e., slips and lapses) and social interactions with patients due to work stress.

Our study suggests a particularly pronounced association of practice organization in terms of working processes and responsibilities for tasks with slips and lapses due to work stress among MAs. Those observations are in line with findings from a cross-sectional, multi-country study suggesting positive associations between organizational/managerial support of nurses and nurses’ self-reported quality of care [29]. Good organizational/managerial support implies nurses’ agreement that the management adequately supports their decisions regarding patient care [29]. A participant observation performed in the UK addressed intravenous medication errors in hospitals and suggested that a lack of knowledge of administrational procedures contributes to those mistakes [30]. In addition, a qualitative study from the UK revealed failures in routine procedures as well as poor adherence to policies to be associated with increased levels of medication errors in hospitals [31]. Empirical evidence specifically for outpatient practices and a link to quality of care is lacking to our knowledge. Taking these and further studies [32,33] into account, we suggest that practice organization may be important to minimize slips and lapses.

In our study, a high workload and poor job control (e.g., interruptions and multitasking) were associated with poor social interaction with patients due to work stress. MAs’ daily work is characterized by a high workload and frequent interruptions while remaining in direct patient contact most of the time [2,3]. A mixed methods study from Belgium found that hospital nurses perceived a high workload and lack of time as barriers to their communication with patients [34]. This is supported by a study that examined communication barriers between nurses and older patients in Korean hospitals [35]. Nurses reported a heavy workload and multitasking to be the most important communication barriers. A high workload and the corresponding time pressure was found to affect communication between nurse and patient negatively [35]. Those studies highlight the link between workload and social interaction with patients, which we also found in our study. In addition, a mixed methods study from Switzerland emphasizes the importance of communication for patient safety, as the quality of telephone triage performed by MAs was described to depend on organizational structures such as job control (disturbances, multitasking) and team collaboration [16].

A further pronounced association we found was existent between poor collaboration and work stress-related poor social interaction with patients among MAs. The work of MAs is highly dependent on the team members in terms of communication, division of labor, and delegation of tasks [2,3,16]. In addition, colleagues are an important source of social support in the health sector [1,3,36,37]. Sturm et al. [38] observed that worse team functioning was associated with higher readmission rates of patients in hospitals. Moreover, studies found that poor collaboration is associated with a poorer communication between health care workers, which has been shown to interfere with the quality of care provided [36,39]. This highlights the importance of collaboration for the delivery of quality of care and is in line with our results.

### 4.2. Potential Pathways

To explain our above-mentioned results, we drew on a theoretical framework that has been suggested for the occupational group of physicians [6,12]. This framework postulates two types of pathways, i.e., direct pathways and indirect effects. The former refers to a system approach, which assumes that the system has inherent weaknesses that manifest themselves in poor work environments [33,40]. The indirect effect involves mediating factors [12] that are caused by poor working conditions and exert themselves adverse effects on the quality of care. Possible mediating factors include, mental health, job satisfaction, and motivation [12].

If applied to our study results, the following direct pathways are conceivable: psychosocial working conditions among MAs, such as a high workload and poor job control, may limit the opportunities or time resources for good communication with patients as well as collaboration within the team, possibly leading to misunderstandings between the MA and the patients as well as colleagues, with potential negative impact on the quality of care [12,36]. A similar mechanism may apply to slips and lapses, as a high workload and low job control may affect concentration and efficacy [34], which may result in slips and lapses. Moreover, poor practice organization may decrease the efficiency of work processes and increase interruptions [41], which in turn could result in more slips and lapses [12,42]. Further, to correct slips and lapses, time is needed, which adds to the experienced time pressure and work stress among MAs [19].

In terms of the indirect pathway the association between high work stress and poor social interaction with patients could be potentially explained by a decreasing job satisfaction and thus reduced commitment and motivation when interacting with patients [12,28]. In addition, work stress may lead to poor mental health among MAs [11], which may result in an increase in slips and lapses [14] as well as poor social interaction [43]. The notion of indirect pathways, e.g., mediation by burnout or absenteeism, is in keeping with findings from different studies [4,10,34].

### 4.3. Methodological Considerations

The study’s cross-sectional design is a limitation, as the direction of association and potential causality remain unknown. Moreover, the number of MAs contacted via our widespread recruitment efforts is not known. Therefore, the response rate cannot be estimated. Possible selection bias cannot be ruled out, as the study may have attracted rather stressed or particularly motivated MAs. However, as detailed in the methods section, the MAs who participated in this study seem to be representative of the MA population in Germany. Another limitation stems from the fact that all variables were measured by participants’ self-reports, which could introduce common method variance: as the majority of our sample expressed high levels of stress according to the ERI ratio, participating MAs might have rated their psychosocial working conditions negatively and also the quality of care indicators to maintain consistency in their response pattern (i.e., consistency motif). This could induce spurious and non-causal associations [44]. However, a cross-sectional study from the Netherlands reported a strong correlation between nurse-reported quality of care and objectively measured quality of care. Nurse-sensitive screening indicators (e.g., standardized pain assessments, malnutrition screening, delirium screening, and observation) were used as objective assessments [45].

The novel and carefully developed instrument used in this study helps to measure impaired quality of care in terms of slips and lapses, and perceived social interaction and can be further used to associate adverse working conditions with quality of care. The comprehensive and careful development process of the instrument in combination with its psychometric properties support its validity for the occupational group of MAs. Furthermore, the questionnaire is short and easy to administer and can serve as a resource for additional research in outpatient settings, such as GP and specialist practices.

### 4.4. Recommendations for Practice, Prevention, and Future Research

Based on our findings, the following recommendations for future research emerge: our results imply that adverse working conditions are associated with a reduced quality of care among MAs. This relationship will need to be verified and clarified in terms of its direction by prospective studies. In order to further explore the validity of our self-developed questionnaire and to substantiate associations found between psychosocial working conditions and quality of care indicators, additional methods for measuring quality of care should be applied in future studies. For instance, studies that rely on observer-based ratings of work processes and interactions in practice consultations, which have already been performed in hospital settings [46,47], could provide more objective assessments of slips and lapses or social interactions with patients among MAs. Further, social interactions could be rated by the patients and could be contrasted with the rating of the corresponding health care professional. These additional measures could improve our understanding of the contexts in which working conditions may affect the quality of care [48] and could provide further evidence on the validity of our questionnaire based on external criteria. Moreover, potential mediators should be examined to better understand the link between psychosocial working conditions and quality of care. Testing of specific pathways was not the aim of this study and is beyond the scope of this report. Additionally, the cross-sectional design of this study limits interpretations on the direction of association and thus also the conclusions drawn for possible mediating effects.

In terms of the development of interventions for daily practice, our findings should be regarded as preliminary due to the cross-sectional study design. However, starting points may be to improve practice organization to decrease slips and lapses. In prior work [49], we suggested structural processes as a promising intervention target: redefining responsibilities as well as clarifying delegation and division of labor could help to reduce and reorganize workload [2,49]. In a review, it was proposed to balance interruptions and patient accessibility to reduce error-producing conditions [50]. Therefore, work (e.g., administrative tasks or answering phone calls) could be decentralized from the reception to more quiet rooms with fewer interruptions. However, that review, which focused on nurses in hospitals, highlighted the difficulty and importance of maintaining this balance and the proposed intervention needs to be tested for feasibility in an ambulatory practice setting [50]. As collaboration is associated with an improved quality of care [38], possible interventions could include regular team meetings or constructive feedback talks with the supervisor. Leadership-development programs could help to improve communication and cooperation across the team and should be extended to MAs and their supervising physicians [51].

## 5. Conclusions

Our study suggests that adverse psychosocial working conditions are associated with frequent self-reported slips and lapses as well as poorer patient interaction due to work stress among MAs. Prospective studies are needed to verify those relationships and to develop suitable interventions to improve the quality of patient care provided by MAs.

## Figures and Tables

**Table 1:** Items and exploratory factor analysis of the self-developed questionnaire measuring quality of care components that medical assistants believe to be affected by work stress.

(Introductory Text): Please Rate Which Areas of Patient Care Are Affected by Your Work Stress.			
Items	Mean (Standard Deviation)	Factor Loading	
		Factor 1: Slips and lapses (Eigenvalue 2.168)	Factor 2: Interaction with patients (Eigenvalue 1.913)
Slips and lapses happen during measurements on patients.	1.78 (0.73)	0.78	
Slips and lapses happen with patient records (mixing up).	1.81 (0.68)	0.88	
Slips and lapses happen during documentation.	1.93 (0.70)	0.87	
I have a hard time being kind to patients.	2.04 (0.88)		0.88
I have less patience with patients.	2.31 (0.91)		0.88
There is too little time to talk to patients.	3.53 (0.95)		0.56

The Eigenvalue represents the total amount of variance of the observed items that is explained by the respective factor. Eigenvalue ≥ 1: the factor explains more variance than the respective variance of the item [24].

**Table 2:** Description of the sample (n * = 887).

Characteristic	
Age, Mean (M) (Standard Deviation (SD))	39.28 (11.43)
Sex, n (%)	
Female	865 (98.41)
Male	14 (1.59)
	M (SD)
Effort	
Score range 6–24, cut-off ≥ 21	18.56 (3.19)
Reward	
Score range 11–44, cut-off ≥ 31	28.25 (5.98)
ERI ratio ^a^	
(Effort * 11)/(reward * 6)	1.28 (0.42)
MA ^b^ sub-scale (high) workload	
Score range 6–24	17.36 (4.19)
MA sub-scale (low) job control	
Score range 6–24	21.11 (2.71)
MA sub-scale (poor) collaboration	
Score range 4–16	8.41 (2.85)
MA sub-scale (low) gratification	
Score range 4–16	11.52 (2.66)
MA sub-scale (poor) practice organization	
Score range 3–12	6.56 (2.08)
MA sub-scale (lack of) resources	
Score range 3–12	4.63 (1.71)
MA sub-scale (poor) leadership behavior	
Score range 3–12	8.01 (2.35)
Slips and lapses ^c^	
Score range 5–15	5.51 (1.79)
Interaction with patients ^c^	
Score range 5–15	7.88 (2.16)
	n (%)
Leadership position (yes)	421 (48.0)
Work stress according to ERI (i.e., ratio >1.0)	616 (73.77)

* n with complete data on the respective variable and item; ^a^ effort-reward imbalance questionnaire (ERI); ^b^ medical assistant (MA); ^c^ questionnaire measuring those quality of care dimensions that medical assistants believe to be affected by their work stress.

**Table 3:** Associations of psychosocial working conditions with slips and lapses or poor interaction with patients due to work stress among medical assistants (Poisson regression).

		Slips and Lapses ^a^				Poor Interaction with Patients ^a^			
Characteristic		Model I ^b^		Model II ^c^		Model I ^b^		Model II ^c^	
		PR	95% CI	PR	95% CI	PR	95% CI	PR	95% CI
ERI model									
Effort	High vs. low ^d^	1.34	1.03, 1.75	1.45	1.10, 1.90	1.87	1.47, 2.38	1.88	1.47, 2.40
Reward	High vs. low	0.51	0.36, 0.71	0.53	0.38, 0.75	0.45	0.33, 0.63	0.42	0.30, 0.59
ERI ratio (>1.0 vs. remainder)	High vs. low	2.46	1.60, 3.78	2.53	1.63, 3.92	3.71	2.31, 5.96	3.62	2.25, 5.81
MA-specific instrument									
Workload (high)	High vs. low	1.58	1.22, 2.05	1.65	1.27, 2.15	2.32	1.83, 2.96	2.33	1.83, 2.98
Job control (low)	High vs. low	1.28	0.99, 1.66	1.48	1.12, 1.94	2.09	1.63, 2.67	2.12	1.65, 2.73
Collaboration (poor)	High vs. low	1.70	1.32, 2.20	1.69	1.30, 2.19	2.02	1.59, 2.57	2.04	1.60, 2.61
Gratification (low)	High vs. low	1.49	1.15, 1.93	1.46	1.12, 1.90	1.33	1.05, 1.70	1.38	1.08, 1.77
Practice organization (poor)	High vs. low	2.28	1.77, 2.93	2.24	1.73, 2.90	1.70	1.34, 2.17	1.74	1.36, 2.23
Resources (lack of)	High vs. low	1.37	1.05, 1.78	1.39	1.07, 1.82	^e^		^e^	
Leadership (poor behavior)	High vs. low	1.24	0.94, 1.63	1.22	0.92, 1.61	1.87	1.47, 1.37	1.94	1.53, 2.48

Effort-reward imbalance questionnaire (ERI) or medical assistant (MA)-specific work stress questionnaire; prevalence ratio (PR) and 95% confidence intervals (95% CIs). ^a^ Outcome variables (score range 3–15) are dichotomized based on highest quintile vs. remaining quintiles. Cut-off ≥ 7 for work stress-associated slips and lapses and ≥ 10 for work stress-associated poor interaction with patients; ^b^ unadjusted; ^c^ additionally adjusted for age, gender, and leadership position; ^d^ all exposure variables are dichotomized based on highest tertile vs. remaining tertiles (high vs. low) except for the ERI ratio; ^e^ sub-scale “resources” removed from analyses for “poor interaction with patients” due to conceptual overlap.

**Table 4:** Sensitivity analysis: associations of psychosocial working conditions (z-scores) with slips and lapse or poor interaction with patients due to work stress among medical assistants (Poisson regression).

		Slips and Lapses ^a^				Poor Interaction with Patients ^a^			
Characteristic		Model I ^b^		Model II ^c^		Model I ^b^		Model II ^c^	
		PR	95% CI	PR	95% CI	PR	95% CI	PR	95% CI
ERI model									
Effort	z-score	1.28	1.11, 1.47	1.33	1.15, 1.53	1.47	1.35, 1.62	1.48	1.35, 1.63
Reward	z-score	0.75	0.66, 0.85	0.75	0.66, 0.86	0.67	0.59, 0.76	0.65	0.57, 0.74
ERI ratio	z-score	1.28	1.16, 1.42	1.28	1.16, 1.42	1.47	1.35, 1.62	1.48	1.35, 1.63
MA-specific instrument									
Workload	z-score	1.37	1.18, 1.58	1.40	1.20, 1.62	1.95	1.69, 2.24	1.95	1.69, 2.25
Job control	z-score	1.20	1.03, 1.40	1.33	1.13, 1.57	1.85	1.56, 2.19	1.89	1.58, 2.26
Collaboration	z-score	1.31	1.16, 1.48	1.30	1.15, 1.47	1.49	1.34, 1.66	1.49	1.34, 1.66
Gratification	z-score	1.32	1.15, 1.50	1.30	1.13, 1.49	1.27	1.13, 1.44	1.32	1.16, 1.49
Practice organization	z-score	1.52	1.36, 1.70	1.50	1.34, 1.68	1.32	1.17, 1.48	1.32	1.17, 1.49
Resources	z-score	1.14	1.01, 1.28	1.13	1.00, 1.28	^d^		^d^	
Leadership	z-score	1.26	1.11, 1.44	1.27	1.11, 1.44	1.50	1.33, 1.69	1.52	1.34, 1.73

Effort-reward imbalance questionnaire (ERI) or medical assistant (MA)-specific work stress questionnaire; prevalence ratio (PR) and 95% confidence intervals (95% CIs). ^a^ Outcome variable is dichotomized based on highest quintile vs. remaining quintiles. Cut-off ≥ 7 for work stress-associated slips and lapses and ≥ 10 for work stress-associated poor interaction with patients; ^b^ unadjusted; ^c^ additionally adjusted for age, gender, and leadership position; ^d^ sub-scale “resources” removed from analyses for “poor interaction with patients” due to conceptual overlaps.

## Data Availability

The data presented in this study are available on request from the corresponding author.

## Supplementary Materials

The following are available online at https://www.mdpi.com/article/10.3390/ijerph18189693/s1: Table S1: Intercorrelation table (Pearson correlation) for dependent and independent variables; Table S2: Associations of psychosocial working conditions with slips and lapses or poor interaction with patients due to work stress among medical assistants (linear regression); Table S3: Associations of psychosocial working conditions (z-scores) with slips and lapse or poor interaction with patients due to work stress among medical assistants (linear regression).
