# Supplementary material for: Associations between Psychosocial Working Conditions and Quality of Care (i.e., Slips and Lapses, and Perceived Social Interactions with Patients)—A Cross-Sectional Study among Medical Assistants
Source: Int J Environ Res Public Health. 2021 Sep 15;18(18):9693. doi: 10.3390/ijerph18189693 (PMC8472247; doi:10.3390/ijerph18189693)
Supplement: Supplementary file 1 [file ijerph-18-09693-s001.zip › ijerph-1318160-supplementary.pdf]

International Journal of
*Environmental Research and Public Health*

MDPI

## Supplementary Material

**Table S1**. Intercorrelation table (Pearson correlation) for dependent and independent variables.

| | SL | IP | Effort | Reward | ERI | WL | JC | CB | GF | PO | RS | LS |
|---|---|---|---|---|---|---|---|---|---|---|---|---|
| SL | 1 | | | | | | | | | | | |
| IP | ,283** | 1 | | | | | | | | | | |
| Effort | ,101** | ,346** | 1 | | | | | | | | | |
| Reward | -,138** | -,277** | -,319** | 1 | | | | | | | | |
| ERI | ,133** | ,349** | ,712** | -,832** | 1 | | | | | | | |
| WL | ,130** | ,392** | ,823** | -,379** | ,638** | 1 | | | | | | |
| JC | ,082* | ,330** | ,747** | -,211** | ,501** | ,591** | 1 | | | | | |
| CB | ,157** | ,310** | ,408** | -,655** | ,662** | ,461** | ,266** | 1 | | | | |
| GF | ,081* | ,186** | ,254** | -,828** | ,652** | ,284** | ,169** | ,393** | 1 | | | |
| PO | ,192** | ,218** | ,253** | -,340** | ,340** | ,361** | ,158** | ,399** | ,152** | 1 | | |
| RS | ,096** | ,356** | 0,052 | -,234** | ,174** | ,100** | -0,004 | ,231** | ,170** | ,177** | 1 | |
| LS | ,112** | ,290** | ,321** | -,649** | ,568** | ,427** | ,244** | ,550** | ,429** | ,506** | ,197** | 1 |

** $p < 0.01$ (two-tailed), * $p < 0.05$ (two-tailed)

n with complete data on the respective variable and item

SL, Slips and lapses; IP, Interaction with patients; ERI, ERI ratio; WL, Workload; JC, Job control; CB, Collaboration; GF, Gratification; PO, Practice organization, RS, Resources; LS, Leadership

**Table S2.** Associations of psychosocial working conditions with slips and lapses or poor interaction with patients due to work stress among medical assistants (linear regression).

| | | Slips and lapses[a] | | | | | | Poor interaction with patients[a] | | | | | |
|---|---|---|---|---|---|---|---|---|---|---|---|---|---|
| Characteristic | | Model I[b] | | | Model II[c] | | | Model I[b] | | | Model II[c] | | |
| | | b | ß | p-value | b | ß | p-value | b | ß | p-value | b | ß | p-value |
| ERI model | | | | | | | | | | | | | |
| Effort (highest tertile vs rest) | High vs low[d] | 0.06 | 0.03 | 0.396 | 0.11 | 0.05 | 0.143 | 0.47 | 0.22 | 0.000 | 0.48 | 0.22 | 0.000 |
| Reward (highest tertile vs rest) | High vs low | -0.25 | -0.12 | 0.001 | -0.23 | -0.11 | 0.002 | -0.49 | -0.24 | 0.000 | -0.52 | -0.25 | 0.000 |
| ERI ratio (>1.0 items vs rest) | High vs low | 0.31 | 0.14 | 0.000 | 0.31 | 0.14 | 0.000 | 0.70 | 0.31 | 0.000 | 0.70 | 0.31 | 0.000 |
| MA-specific instrument | | | | | | | | | | | | | |
| Workload (high) | High vs low | 0.18 | 0.08 | 0.014 | 0.20 | 0.10 | 0.006 | 0.56 | 0.27 | 0.000 | 0.56 | 0.27 | 0.000 |
| Job control (low) | High vs low | 0.07 | 0.03 | 0.348 | 0.14 | 0.07 | 0.061 | 0.50 | 0.25 | 0.000 | 0.52 | 0.25 | 0.000 |
| Collaboration (poor) | High vs low | 0.22 | 0.10 | 0.002 | 0.22 | 0.10 | 0.002 | 0.47 | 0.22 | 0.000 | 0.47 | 0.22 | 0.000 |
| Gratification (low) | High vs low | 0.08 | 0.04 | 0.274 | 0.06 | 0.03 | 0.389 | 0.26 | 0.13 | 0.000 | 0.28 | 0.14 | 0.000 |
| Practice organization (poor) | High vs low | 0.38 | 0.18 | 0.000 | 0.39 | 0.18 | 0.000 | 0.37 | 0.17 | 0.000 | 0.39 | 0.18 | 0.000 |
| Resources (lack of) | High vs low | 0.28 | 0.13 | 0.000 | 0.30 | 0.14 | 0.000 | [e] | | | [e] | | |
| Leadership (poor behavior) | High vs low | 0.12 | 0.05 | 0.132 | 0.11 | 0.05 | 0.163 | 0.43 | 0.19 | 0.000 | 0.44 | 0.20 | 0.000 |

Effort-reward imbalance questionnaire (ERI) or medical assistant (MA)-specific work stress questionnaire
Quality of care MA-specific questionnaire
[a] Outcome variables used as z-scores
[b] Unadjusted
[c] Additionally adjusted for age, gender and leadership position
[d] Exposure variables are dichotomized based on highest tertile vs remaining tertiles (high vs. low)
[e] Subscale "resources" removed from analyses for "poor interaction with patients" due to conceptual overlaps

**Table S3.** Associations of psychosocial working conditions (z-scores) with slips and lapse or poor interaction with patients due to work stress among medical assistants (linear regression).

| | | Slips and lapses[a] | | | | | | Poor interaction with patients[a] | | | | | |
|---|---|---|---|---|---|---|---|---|---|---|---|---|---|
| Characteristic | | Model I[b] | | | Model II[c] | | | Model I[b] | | | Model II[c] | | |
| | | b | ß | p-value | b | ß | p-value | b | ß | p-value | b | ß | p-value |
| ERI model | | | | | | | | | | | | | |
| Effort (highest tertile vs rest) | z-score | 0.10 | 0.10 | 0.003 | 0.12 | 0.12 | 0.000 | 0.35 | 0.35 | 0.000 | 0.35 | 0.35 | 0.000 |
| Reward (highest tertile vs rest) | z-score | - 0.14 | - 0.14 | 0.000 | - 0.13 | - 0.13 | 0.000 | - 0.28 | - 0.28 | 0.000 | - 0.29 | - 0.29 | 0.000 |
| ERI ratio (>1.0 items vs rest) | z-score | 0.13 | 0.13 | 0.000 | 0.14 | 0.14 | 0.000 | 0.35 | 0.35 | 0.000 | 0.35 | 0.35 | 0.000 |
| MA-specific instrument | | | | | | | | | | | | | |
| Workload (high) | z-score | 0.13 | 0.13 | 0.000 | 0.14 | 0.14 | 0.000 | 0.39 | 0.39 | 0.000 | 0.39 | 0.39 | 0.000 |
| Job control (low) | z-score | 0.08 | 0.08 | 0.015 | 0.13 | 0.13 | 0.000 | 0.33 | 0.33 | 0.000 | 0.34 | 0.34 | 0.000 |
| Collaboration (poor) | z-score | 0.16 | 0.16 | 0.000 | 0.16 | 0.16 | 0.000 | 0.31 | 0.31 | 0.000 | 0.31 | 0.31 | 0.000 |
| Gratification (low) | z-score | 0.08 | 0.08 | 0.017 | 0.07 | 0.07 | 0.034 | 0.19 | 0.19 | 0.000 | 0.20 | 0.20 | 0.000 |
| Practice organization (poor) | z-score | 0.19 | 0.19 | 0.000 | 0.19 | 0.19 | 0.000 | 0.22 | 0.22 | 0.000 | 0.23 | 0.23 | 0.000 |
| Resources (lack of) | z-score | 0.10 | 0.10 | 0.005 | 0.10 | 0.10 | 0.004 | [d] | | | [d] | | |
| Leadership (poor behavior) | z-score | 0.11 | 0.11 | 0.001 | 0.12 | 0.12 | 0.001 | 0.29 | 0.29 | 0.000 | 0.30 | 0.30 | 0.000 |

Effort-reward imbalance questionnaire (ERI) or medical assistant (MA)-specific work stress questionnaire
Quality of care MA-specific questionnaire
[a] Outcome variables used as z-scores
[b] Unadjusted
[c] Additionally adjusted for age, gender and leadership position
[d] Subscale "resources" removed from analyses for "poor interaction with patients" due to conceptual overlaps
